# Don’t Blame Bactrim: A Case Report of RIME (Reactive Infectious Mucocutaneous Eruption) in Disguise

**DOI:** 10.51894/001c.144427

**Published:** 2025-09-30

**Authors:** Sweta Banda

**Affiliations:** 1 Department of Emergency Medicine Henry Ford Wyandotte

## 67

### INTRODUCTION

Reactive Infectious Mucocutaneous Eruption (RIME) is a rare and increasingly recognized condition characterized by mucosal involvement following respiratory infections, predominantly affecting children and adolescents. In contrast to other etiologies of mucosal rash, such as Stevens-Johnson Syndrome (SJS) or Toxic Epidermal Necrolysis (TEN), RIME manifests with relatively milder mucosal symptoms, including oral, ocular, and genital erosions, and typically follows a more favorable clinical trajectory.

### CASE DESCRIPTION

This case report describes a 23-year-old female who developed RIME following an influenza A infection and subsequent exposure to Bactrim. The patient presented with bilateral conjunctivitis, oral ulcerations, vaginal lesions, and vaginal discharge, emerging seven days after the onset of influenza symptoms and five days after initiating Bactrim therapy for a urinary tract infection (UTI). Laboratory testing confirmed an active influenza A infection, and a clinical diagnosis of RIME was established following comprehensive dermatologic and ophthalmologic evaluation.

### DISCUSSION/CONCLUSIONS

In the emergency department, Stevens-Johnson Syndrome (SJS) represents the most life-threatening etiology of mucosal rash following Bactrim exposure. In this case, the patient’s symptoms emerged in a sequential pattern after influenza infection and subsequent Bactrim administration, emphasizing the infection-related pathogenesis of mucocutaneous involvement. The pathophysiology of Reactive Infectious Mucocutaneous Eruption (RIME) is thought to involve an immune-mediated response to bacterial or viral infections, resulting in mucosal erosions without significant cutaneous involvement.

Management in this case was primarily supportive, including oral corticosteroids, ciprofloxacin for the urinary tract infection, and analgesia. The patient exhibited a favorable response to treatment, reinforcing the generally benign course of RIME in contrast to other severe mucocutaneous disorders.

This case highlights the necessity of considering RIME in the differential diagnosis of mucosal lesions following respiratory infections, particularly in the absence of widespread cutaneous involvement. Early recognition and appropriate intervention are critical to preventing complications and optimizing patient outcomes. Further research is warranted to enhance diagnostic accuracy and refine therapeutic strategies for this emerging condition.

